# Reduced Antibodies and Innate Cytokine Changes in SARS-CoV-2 BNT162b2 mRNA Vaccinated Transplant Patients With Hematological Malignancies

**DOI:** 10.3389/fimmu.2022.899972

**Published:** 2022-05-25

**Authors:** Cristina Bergamaschi, Maria Pagoni, Margherita Rosati, Matthew Angel, Ifigeneia Tzannou, Margarita Vlachou, Ismini Darmani, Amirah Ullah, Jenifer Bear, Santhi Devasundaram, Robert Burns, Ioannis Baltadakis, Stavros Gigantes, Meletios-Athanasios Dimopoulos, George N. Pavlakis, Evangelos Terpos, Barbara K. Felber

**Affiliations:** ^1^Human Retrovirus Pathogenesis Section, Vaccine Branch, Center for Cancer Research, National Cancer Institute at Frederick, Frederick, MD, United States; ^2^Department of Hematology & Lymphomas and Bone Marrow Transplantation Unit, Evangelismos General Hospital, Athens, Greece; ^3^Human Retrovirus Section, Vaccine Branch, Center for Cancer Research, National Cancer Institute at Frederick, Frederick, MD, United States; ^4^Vaccine Branch, Center for Cancer Research, National Cancer Institute, Bethesda, MD, United States; ^5^Center for Cancer Research Collaborative Bioinformatics Resource, Leidos Biomedical Research, Inc., Frederick National Laboratory for Cancer Research, Frederick, MD, United States; ^6^Pharmacy Department, Evangelismos General Hospital, Athens, Greece; ^7^Department of Clinical Therapeutics, School of Medicine, National and Kapodistrian University of Athens, Athens, Greece

**Keywords:** humoral response, cytokine, SARS-CoV-2 BNT162b2 mRNA vaccine, IFN-gamma, CXCL10, IL-15, transplantation, hematological cancer

## Abstract

Immunocompromised individuals including patients with hematological malignancies constitute a population at high risk of developing severe disease upon SARS-CoV-2 infection. Protection afforded by vaccination is frequently low and the biology leading to altered vaccine efficacy is not fully understood. A patient cohort who had received bone marrow transplantation or CAR-T cells was studied following a 2-dose BNT162b2 mRNA vaccination and compared to healthy vaccine recipients. Anti-Spike antibody and systemic innate responses were compared in the two vaccine cohorts. The patients had significantly lower SARS-CoV-2 Spike antibodies to the Wuhan strain, with proportional lower cross-recognition of Beta, Delta, and Omicron Spike-RBD proteins. Both cohorts neutralized the wildtype WA1 and Delta but not Omicron. Vaccination elicited an innate cytokine signature featuring IFN-γ, IL-15 and IP-10/CXCL10, but most patients showed a diminished systemic cytokine response. In patients who failed to develop antibodies, the innate systemic response was dominated by IL-8 and MIP-1α with significant attenuation in the IFN-γ, IL-15 and IP-10/CXCL10 signature response. Changes in IFN-γ and IP-10/CXCL10 at priming vaccination and IFN-γ, IL-15, IL-7 and IL-10 upon booster vaccination correlated with the Spike antibody magnitude and were predictive of successful antibody development. Overall, the patients showed heterogeneous adaptive and innate responses with lower humoral and reduced innate cytokine responses to vaccination compared to naïve vaccine recipients. The pattern of responses described offer novel prognostic approaches for potentiating the effectiveness of COVID-19 vaccination in transplant patients with hematological malignancies.

## Introduction

The introduction of vaccines against SARS-CoV-2 has resulted in a significant reduction of COVID-19 associated severe disease and deaths. However, vaccination efficacy is impaired in immunocompromised individuals, including patients with malignancies. Several studies have investigated both safety and immunogenicity of COVID-19 vaccines in patients with different hematological cancers (1) [reviewed in (2)]. Therapy-related immunosuppression and disease-related immune dysregulation with alterations in both B and T cell compartments contribute to the low humoral response (3–18). In addition, anti-cancer therapies such as chemotherapy, anti-CD20 antibodies, bone marrow transplantation, cell transfer (e.g., CAR-T cells) following lymphodepleting pre-conditioning, further weaken the ability of the immune system to mount effective adaptive responses. Patients with hematological malignancies who receive allogeneic stem cell transplantation (AlloSCT) appear to have altered humoral immunity after vaccination. Patients who are receiving immunosuppressive therapy demonstrate an inadequate humoral response to the BNT162b2 vaccine, while recipients who are off immunosuppression tend to have a humoral response comparable to that of the general population (3, 16, 19). However, the biology behind the efficacy of BNT162b2 vaccine in these patients is not fully understood and there is no data in the literature for the efficacy of the vaccine against variants of concern (VOC), such as the highly infectious and pathogenic Delta variant and the recent highly contagious Omicron variant of SARS-CoV-2.

Cytokines and chemokines play an important role in shaping adaptive immunity in response to infection and vaccination. Several reports have described the immune signatures associated with Yellow Fever, HIV-Ade5, HIV ALVAC, and COVID-19 mRNA-based vaccines (20–25). We have previously reported that BNT162b2 mRNA vaccination in both COVID-19-naive and in previously infected health care workers (HCW) induced a systemic cytokine/chemokine signature featuring IL-15, IFN-γ, and IP-10/CXCL10 (21). Importantly, increased expression of IFN-γ and IL-15 correlated with higher antibody (Ab) titers against SARS-CoV-2 Spike, suggesting their potential use as biomarkers of efficient humoral immunity development in response to vaccination.

Considering the low seroconversion rate upon COVID-19 vaccination in patients with hematological malignancies subjected to stem cell transplantation (SCT), there is a critical need to develop optimal strategies for improved protection. In particular, the characterization of systemic responses to vaccination could allow stratification of patients and identification of biomarkers predictive of effective development of vaccine-induced humoral responses.

In this study, we determined differences in anti-SARS-CoV-2 Spike Ab titers and the cytokine signature elicited upon BNT162b2 mRNA vaccination, comparing a cohort of hematological transplant patients to a cohort of COVID-19-naive health care workers (HCW), as control vaccine recipients. The responses were analyzed after the 1^st^ and 2^nd^ vaccination, and due to the circumstances, we could not examine this patient cohort after the 3^rd^ vaccination.

## Materials and Methods

### Patients and Controls

This is a prospective study that was designed to determine the kinetics of anti-SARS-CoV-2 Ab after immunization with the BNT162b2 mRNA vaccine (NCT04743388) (**Supplementary Table 1**). Major inclusion criteria for the transplant study included: (i) age above 18 years; (ii) receipt of allogeneic or autologous SCT or receipt of CAR-T cell immunotherapy for hematological malignancy and (iii) eligibility for vaccination (>3 months from the time of transplant or cellular therapy and absence of uncontrolled graft-versus-host disease). Data from naïve health care workers (HCW) vaccine recipients, of similar age and gender, who were vaccinated during the same time period (January-May 2021) were also included in this analysis.

Major exclusion criteria for both patients and controls included the presence of: (i) autoimmune disorders or active malignant disease besides the hematological malignancy; (ii) HIV or active hepatitis B and C infection and (iii) end-stage renal disease. These disease entities were excluded due to concerns of confounding effect on Ab response following vaccination. Relevant data were extracted from the medical records and included: demographics, complete blood count, disease status, type, and time of treatment.

βlood collection schedules were as follows: on day 1, just before the first vaccination dose, on day 2, on day 22 (the day of the second vaccination and just before receiving the injection), on day 23, and on day 50 (four weeks post the 2^nd^ vaccine shot). Blood was drawn and serum was isolated within 4 hrs of collection. The serum was then refrigerated at -80°C until the day of measurement.

According to the National Vaccination Program in Greece, the two doses of BNT162b2 were administered three weeks apart.

### SARS-CoV-2 Antibody Measurements

In-house ELISA assays using a panel of purified trimeric complete Wuhan strain (WA1) Spike and Spike-RBD and a panel of VOC Spike-RBD proteins (Beta B.1.351, Delta B.1.617.2, Omicron B.1.1.529) were detailed elsewhere (26–28). Ab levels were measured using eight 4-fold serial serum dilutions starting at 1:50 and endpoint titers were determined using lastX feature using GraphPad prism area-under-the curve program. The ELISA was tested against the WHO reference panel of SARS-CoV-2 immunoglobulin (NIBSC code 20/268). Equation for conversion of the in-house ELISA endpoint titers to WHO international standard (BAU/ml): Spike Y = 0.004947*X + 29.09; Spike-RBD Y = 0.02357*X + 19.55 (Y=BAU/ml) with X=endpoint titer.

Neutralization was performed using a pseudotyped HIV_NL_ΔEnv-Nanoluc assay (29, 30) carrying WA1 Spike or Delta Spike proteins (AA 1-1254) as described (27, 31, 32). The highest serum concentration analyzed was a 1:40 dilution and a four-fold serial dilution up to 1:655,360 was tested. Two days later, the luciferase levels were measured in the cell extracts as ID50 (50% Inhibitory Dose) calculated using GraphPad Prism version 9.2 for MacOS X (GraphPad Software, Inc, La Jolla, CA). The NAb ID50 threshold of quantification in this assay is 0.5 log and the threshold of detection is 0.1 log. The NAb assay was tested against the WHO reference panel of SARS-CoV-2 immunoglobulin (NIBSC code 20/268). Equation for conversion of the in-house NAb ID50 titer to WHO international standard (IU/ml): Y = 0.3987*X + 1.374 with X=NAb titer.

### Cytokine/Chemokine Analysis

Serum cytokine/chemokine concentrations were measured with the V-PLEX Human Biomarker Assay kit (Meso Scale Diagnostics, Maryland, US) according to manufacturer’s recommendations. This allowed for the concurrent measurement of 47 cytokines and chemokines (**Supplementary Table 2**). For analysis, biomarkers falling below the detection limit/standard range were removed if absent in more than 50% of the samples or adjusted to 0.5 of the lowest standard point/detection limits.

### Bioinformatics

Biomarker analysis was performed with a workflow written in R using RStudio. The limma package was used to compare biomarker changes between timepoints, setting significance for adjusted p value <0.05. Heatmaps were represented as log_2_ fold change over day 1 (1^st^ vaccination) or day 22 (2^nd^ vaccination) with the corrplot r package (v0.91). Pairwise correlations were performed among the log_2_ fold-changes for measurable sera mediators, using an adjusted Spearman p value <0.05. Cytokine network was generated using Graphia with edges representing a Spearman correlation coefficient greater than 0.2 and k-NN edge reduction of 3, according to Thwaites, et al. (33).

Analysis was deposited at link: https://github.com/NCI-VB/bergamaschi_pfizer_cancer.

### Statistical Analysis

This work analyzed samples from 29 patients and 57 naïve individuals who received two doses of SARS-CoV-2 BNT162b2 mRNA vaccine and from whom blood samples were collected at day 1, 2, 22, 23 during and after the 1^st^ and 2^nd^ vaccination, respectively, which were the criteria to be included in this study. All patients accepting to donate samples were included. The cohort of 57 naïve vaccinees (HCW), not previously exposed to SARS-CoV-2, has been reported previously (21). No prior sample size determination was performed since there was no known properties of the patient cohort related to COVID-19 vaccination. There was no randomization performed due to the nature of the study. The inclusion/exclusion criteria for transplantation patients are described above, although they are not central to the manuscript, which is to compare the development of immune response to vaccination of the two cohorts.

**Figure 1:**
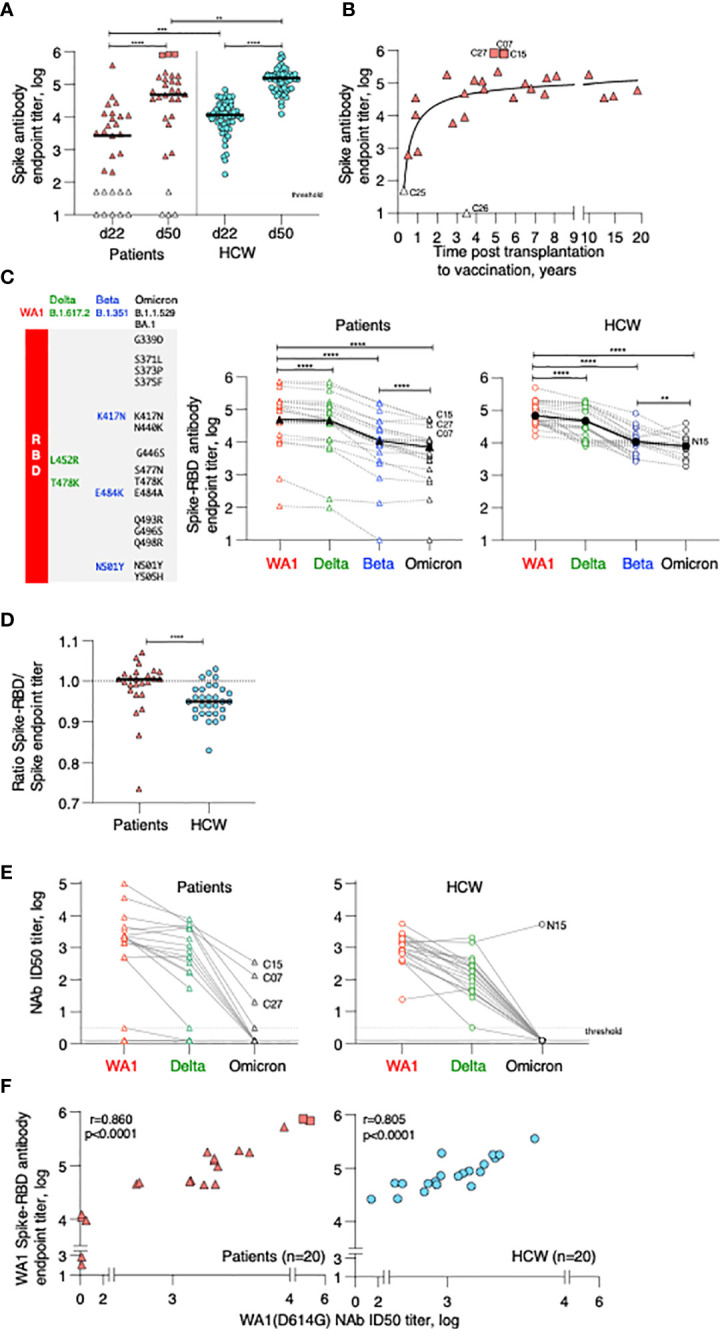
Anti-Spike antibody in patients and control BNT162b2 mRNA vaccine recipients. **(A)** Endpoint titers (log) of wildtype Wuhan (WA1) Spike Ab measured by ELISA at d22 (day of 2^nd^ dose) and d50 post vaccination (one month later). The two cohorts are denoted as patient (triangle symbols; n = 29) and HCW vaccine recipients (circle symbols; n = 55, analyzed at both timepoints). Patients with high titers (C07, C15, C27; square symbols) and antibody-negative (open triangles: C16, C18) received CAR-T cells; C25, C26 received treatment affecting B cell development and function are indicated. A negative titer is entered as 1 log, a titer at the threshold of detection is entered as 1.7 log. The p values are from Wilcoxon test for same-group comparison and Mann-Whitney for comparison of the same timepoint between the two groups. **(B)** Association of anti-Spike Ab titers and time (years) between transplantation and vaccination (n = 27). **(C)** Cartoon illustrating amino acid changes in the Spike-Receptor Binding Domain (RBD) of the VOC Spike variants Delta, Beta and Omicron (left panel). Anti-Spike Ab endpoint titers measured by ELISA against the panel of Spike-RBD at d50 in the vaccinated patients (middle panel, n = 25) and in a subset of HCW (right panel; n = 30 with n = 19 for Omicron Spike-RBD). p values are from paired Wilcoxon test. Black solid symbols and line denotes median. **(D)** Ratio of Spike-RBD/Spike Ab titers of patients (n = 25) and HCW (n = 30) at d50. **(E)** Neutralization was performed using a pseudotyped HIV_NL_ ΔEnv-Nanoluc assay carrying a panel of Spike (AA 1–1254) proteins (WA1, Delta, Omicron). Infectious dose 50 (ID50) values are plotted in log. Patients were analyzed for WA1 and Delta (20 of 25, due to sample availability) and a subset of patients (n = 10) were further analyzed for Omicron (sample availability and/or positivity for WA1 response). A subset of HCW was analyzed for WA1 and Delta (n = 20), of which 19, due to sample availability, were analyzed for Omicron. **(F)** Correlation of WA1(D614G) NAb ID50 titers (log) and WA1 anti-Spike-RBD endpoint titers (log) of patients (triangles) and HCW (circles) vaccine recipients at d50. Spearman r and p values are indicated. The p values are indicated as **, <0.01; ***, <0.001; ****, <0.0001.

For the day 50 analysis of Ab responses, samples from all 29 patients and 55 of 57 naïve individuals were collected. Detailed follow-up Ab analysis was not performed on samples at or below threshold of detection as determined in **Figure 1A** or due to limited sample volume. Statistical analysis (**Figures 1**, **5**, **7**, **S1**, **S4**) was performed with the GraphPad Prism 9.2.0 Software for mac using GraphPad Software, San Diego, California USA, www.graphpad.com. For same-group comparisons, the p values are from non-parametric Wilcoxon test. For comparisons of the same timepoint between the two cohorts, the p values are from non-parametric Mann-Whitney test. Non-parametric Spearman correlations were performed. A value p < 0.05 was considered statistically significant (*, <0.05; **<0.01; ***<0.001; ****<0.0001).

For cytokine analysis, biomarkers falling below the detection limit/standard range were removed if absent in more than 50% of the samples or adjusted to 0.5 of the lowest standard point/detection limits. Twenty-nine analytes were above the threshold and included in the analysis. Bioinformatics analysis was performed using the software: R (https://www.r-project.org/v3.5.1); limma package (https://bioconductor.org/packages/release/bioc/html/limma.html v3.38.3); corrplot r package (https://cran.r-project.org/web/packages/corrplot/index.html v0.84); and Graphia, as described in the Bioinformatics section. Differences in sera cytokine levels at each timepoint between patient and HCW cohorts were analyzed using Mann-Whitney test (GraphPad Software).

The data of the four patients with Ab responses at or below threshold of detection (termed low responders) were excluded (**Figures 3** and **7**; sample size n=25) or shown in addition in separate panels (**Figure 5**, **S2**; **S4**) as indicated in the respective Figures and Figure legends.

## Results

### Patients’ Characteristics

A total of 29 patients with hematologic malignancies were evaluated upon receiving the SARS-CoV-2 BNT162b2 mRNA vaccine. All patients had received cellular therapy at the BMT Unit of Evangelismos General Hospital at a median of 4.4 years (range 0.3-19.3 years) before vaccination (**Table 1**). None of the patients had a history of SARS-CoV-2 infection. They were screened by PCR and anti-Spike Ab by ELISA and were negative for COVID-19 at study entry. They received two doses of the BNT162b2 (Pfizer/BioNtech) mRNA vaccine administered at days 1 and 22. The patient demographic shows 11 male patients (37.9%) and 18 female patients (62.1%) (**Table 1**). The median age was 46 years (range 30-68 years). The cohort showed heterogenous diagnosis with plurality of the patients diagnosed with acute myeloid leukemia (44.8%, 13 patients), followed by non-Hodgkin lymphoma (13.8%, 4 patients), acute lymphocytic leukemia (10.3%, 3 patients), myelodysplastic syndrome (6.9%, 2 patients), Hodgkin lymphoma (6.9%, 2 patients), myelofibrosis (3.4%, 1 patient), chronic lymphocytic leukemia (3.4%, 1 patient), chronic myeloid leukemia (3.4%, 1 patient), mycosis fungoides (3.4%, 1 patient) and multiple myeloma (3.4%, 1 patient). Of the 29 patients, 25 had undergone allogeneic SCT (86.2%) of which 2 patients received an autologous SCT followed by allogeneic SCT; 2 patients received autologous SCT; 2 patients received CAR-T cells of which one had received prior autologous SCT (**Supplementary Table 1**).

**Table 1:** Cohort demographics.

Summary		Patient vaccine recipients	Naïve vaccine recipients (HCW)^1^
n (total)		29	57
Gender	Male n (%)	11 (37.9%)	23 (40.4%)
	Female n (%)	18 (62.1%)	34 (59.6%)
Age	Median (range)	46 (30-68)	51 (28-68)
<50 years	n (%)	17 (58.6%)	28 (49%)
>50 years	n (%)	12 (41.4%)	29 (51%)
Time: transplantation to vaccination	Median (range)	4.4 yrs (0.3-19.3)	not applicable

^1^from Bergamaschi et al, Cell Rep: 36:109504; 2021.

The patient cohort was compared to a cohort of SARS-CoV-2 negative, healthy hospital care workers (HCW) who had received two BNT162b2 mRNA vaccinations and served as controls (n=57). They were screened by PCR and anti-Spike Ab by ELISA and only COVID-19 negative persons were included in this study. This cohort showed similar age and gender distribution as the patient cohort (**Table 1**) (21).

### Distinct Anti-Spike Antibody Responses to BNT162b2 mRNA Vaccine in Post-Cellular Therapy Patients and HCW

The development of anti-Spike Ab was monitored in serum samples collected on day 22 (d22; before administration of the 2^nd^ dose) and on day 50 (d50; one month after the 2^nd^ dose) using an in-house ELISA measuring binding to the trimeric wildtype Wuhan (WA1) Spike. On d22, 3 weeks after the 1^st^ vaccination, the patient cohort showed a wide range of anti-Spike Ab titers (median 3.4 log, range undetected to 5.6 log). Although responses significantly increased upon the 2^nd^ vaccination, Ab titers maintained a wide range (median 4.7 log, range undetected to 5.9 log) (**Figure 1A**). We noticed 3 patients with very high responses (C07, C15, C27; square symbols), 22 patients with intermediate range of responses (triangles) and 4 patients remaining negative (at or below threshold of detection, termed low responders; open triangles). Of these anti-Spike Ab negative patients, two (C16, C18) had received CAR-T cell therapy and two (C25, C26) had received allogeneic transplantation and are on current concomitant medication (steroids and ruxolitinib) known to affect B cell development and function and lacked B cells (**Supplementary Table 1**). To evaluate effects of the transplantation on humoral immune response development, we found a non-linear relation between anti-Spike Ab titers and time of therapy (**Figure 1B**). Although vaccination at less than one year post transplantation resulted in the development of anti-Spike Ab, increasing time from treatment (~2 years) resulted in higher adaptive immune responses.

Comparison of anti-Spike Ab levels between the patient cohort (n=29) and the naïve BNT162b2 mRNA vaccine recipients (HCW, n=57) (**Figure 1A**) showed significantly lower levels in the patient cohort, both at d22 and d50. Similar data were obtained using a subgroup of HCW (n=30 or 20) (**Supplementary Figure 1A**), used for further analysis **(**see below, **Figures 1C, E, F**). Ab levels at d22 directly correlated with levels at d50, and thus, the d22 levels were predictive of the responses found upon the 2^nd^ vaccination (**Supplementary Figure 1B**). The anti-Spike Ab induced in the patient cohort also potently recognized a panel of Spike-RBD proteins from variants of concern (VOC) (**Figure 1C**, left panel) which differ in 2, 3 and 15 AA, respectively, from the wildtype WA1 receptor binding domain (RBD). In comparison to WA1, significantly lower binding was found to Delta, Beta and Omicron (**Figure 1C**, middle and right panels). There is a significant weaker binding to Omicron Spike-RBD, due to distinct additional AA changes in RBD negatively affecting recognition by the WA1 vaccine-induced anti-RBD Ab. The three patients with the highest WA1 Ab also ranked highest against the VOC Spike-RBD. A similar ranking of responses to VOC Spike-RBD was found for the HCW cohort (**Figure 1C**, right panel).

Despite differences in Ab titers recognizing the complete Spike (**Figure 1A**), we found similar levels of recognition of the different Spike-RBD proteins among the two cohorts (**Supplementary Figure 1C**). We further noted that there is a significant difference in the ratio of Spike-RBD/Spike Ab titers comparing patients versus HCW (**Figure 1D**). These data indicate that while HCW develop Ab to different portions of Spike, in contrast, this patient cohort developed Ab recognizing primarily the more dominant RBD. These data explain the apparent discrepancy between Ab responses to complete Spike and Spike-RBD comparing patient and HCW cohorts.

Antibodies were evaluated for their ability to neutralize pseudotyped HIV-derived viruses each carrying WA1, Delta, and Omicron Spike, respectively. Both cohorts showed robust neutralization of WA1 and Delta Spike pseudotyped viruses (**Figure 1D**) with significant correlations between NAb and Spike-RBD Ab titers targeting WA1 (**Figure 1E**). Similar strong correlations were found between NAb and RBD Ab titers targeting Delta, although the NAb and Ab titers were lower in both cohorts (**Supplementary Figure 1B**). NAb induced by the WA1 mRNA vaccine also directly correlated with the ability to neutralize the Delta variant (**Supplementary Figure 1C**). The strong neutralization of WA1 and Delta pseudotyped viruses was contrasted by loss of neutralization of the Omicron pseudotyped virus in most vaccinees of both cohorts (**Figure 1D**). The vaccinees who showed Omicron neutralization were the three patients with the highest Ab responses (C07, C15, C27) and one control vaccinee (N15). Thus, binding of the vaccine-induced anti-Spike Ab to Omicron (**Figure 1C**) did not translate to neutralization of such pseudotyped viruses (**Figure 1D**). Four of the patients were subsequently infected by SARS-CoV-2 (**Supplementary Table 1**) at 4-9 months post vaccination. Two (C03, C05) had good NAb against Delta (ID50 3.6 and 2.5 log, respectively), whereas the other two (C18, C26) did not develop anti-Spike Ab (**Supplementary Table 1**).

In conclusion, these data showed a significant difference in the magnitude of anti-Spike Ab titers between the two cohorts, with patients who underwent cell therapies for hematological malignancies showing lower Ab titers and a proportional lower ability of cross-recognition of different Spike-RBD proteins but maintaining the ability to neutralize wildtype WA1 and the Delta variant. The great differences to Omicron Spike within the RBD resulted in significant lower binding and in poor neutralization in both cohorts.

### Cytokine Profile Induced by the BNT162b2 mRNA Vaccine in Patients

To determine the innate signature induced by BNT162b2 mRNA vaccination in patients, we analyzed the serum cytokine levels using the Meso Scale Discovery (MSD) platform on the day of and at 24 hrs after the 1^st^ and the 2^nd^ vaccination (d1, d2, and d22, d23, respectively). Determination for 47 analytes was performed, of which 29 were above the threshold of detection (**Supplementary Table 2**).

**Figure 2:**
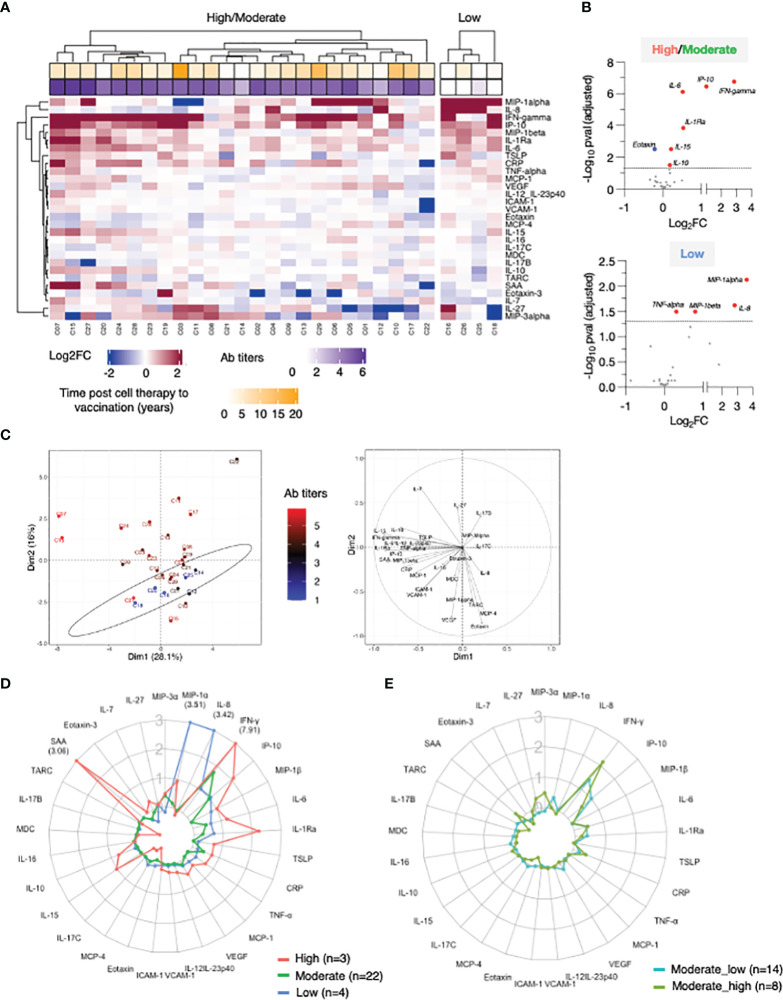
Cytokine profile in patients after 2^nd^ vaccination. Serum levels were measured before (d22) and 24 hours (d23) after 2^nd^ vaccination using the MSD platform for patients. **(A)** Heatmap depicted log2FC (d23_d22) in 29 analytes. Heatmap is annotated with anti-Spike Ab titers measured at d50 and years since cell therapy (transplantation and CAR-T). Patients were separated int two groups based on their ability to mount anti-Spike Ab responses to the vaccination: high/moderate responders (left heatmap) and non-responders (right heatmap). Patients are clustered based on their overall cytokine/chemokine profile. **(B)** Volcano plot of data shown in Panel A depicts differentially expressed cytokines upon 2^nd^ vaccination at d23 in comparison to d22. Vaccine-induced effects are shown for high responders (left panel) and non-responders (right panel). Red dots indicate significant upregulation; blue dots indicate significant downregulation (adjusted p value<0.05 represented by the broken horizontal line). **(C)** PCA plot (left panel) distinguishes patients based on their systemic cytokine responses to the 2^nd^ vaccination, capturing 28.1% and 16% of the dataset variance along PC1 and PC2, respectively. Patients are annotated with their anti-Spike Ab titers measured at d50, with the highest value in red and lowest in blue. PCA loading plot (right panel) showed how strongly cytokines (depicted as grey vectors) influence both PC1 and PC2. The projection of the vectors on PC1 and PC2 corresponds to the loading value (weight) of the cytokine they represent. **(D)** Patients were separated into 3 groups based on anti-Spike antibody titers measured at d50 (low in blue, n = 4; moderate in green, n = 22, green; high in red, n = 3, red). Radar plot compared the median log2FC for each cytokine after 2^nd^ vaccination (d23_22) among the 3 groups. Plot axis is truncated at log2FC=3, with values exceeding this cutoff shown in parentheses (high responders: IFN-γ=7.91; SAA=3.06). **(E)** Patients in the moderate group were further separated into moderate_high (n=8) and moderate_low (n = 14). Radar plot compared the median log2FC for each cytokine after 2^nd^ vaccination (d23_22) between the 2 subgroups.

The 2^nd^ vaccination resulted in the most significant changes in systemic cytokine levels, as we previously reported for the HCW cohort (21). The heatmaps (**Figure 2A**) showed the cytokine log2 fold changes (log2FC) at 24 hours after the 2^nd^ vaccination, annotated with patient information regarding the levels of anti-Spike Ab titers measured at d50 and the time between cell therapy (transplantation and CAR-T) and vaccination. Patients were sub-grouped into high/moderate responders (n=25) and low responders (n=4), based on their ability to mount humoral responses after vaccination (**Figure 1A**).

IFN-γ, IP-10/CXCL10, MIP-1β, IL-1Ra, IL-6, and CRP showed the strongest vaccine-induced upregulation, detected in most patients. Other cytokines showed more patients-to-patients variability. Induction of IL-15, IL-10, IL-7, SAA, MCP-1, TNF-α, and IL-12/IL-23p40 was stronger in patients who developed higher Ab titers (**Figure 2A**). The three patients (C07, C15 and C27; square symbol; **Figure 1A**, left panel) who mounted the highest levels of anti-Spike Ab clustered together and showed the strongest innate signature upon the 2^nd^ vaccine dose. Patients with intermediate and no anti-Spike Ab responses differed as the effects on several cytokines were diminished or absent. The four patients (C16, C26, C25, and C18) who failed to mount a detectable humoral response to vaccination presented a cytokine profile characterized by the strongest upregulation of MIP-1α and IL-8. Downregulation of Eotaxin, MCP-4, IL-17 family of cytokines and TARC was also observed, mainly in patients in the high/moderate responder group (**Figure 2A**).

Differential expression analysis was performed in both groups of patients (**Figure 2B**). In the high/moderate responders (**Figure 2B**, top panel), IFN-γ was the most significantly upregulated cytokine upon 2^nd^ vaccination (~6-fold). The cytokines IP-10/CXCL10, IL-6, IL-1Ra, IL-15, and IL-10 also showed significant (1.5 and 2-fold) upregulations. Eotaxin showed significant downregulation at d23, as previously reported (21). In patients with low/no anti-Spike Ab titers (**Figure 2B**, bottom panel) significant increases were observed in MIP-1α, IL-8, MIP-1β and TNF-α.

Principal Component Analysis (PCA) was used to determine the variance among patients, using the cytokine log2FC upon 2^nd^ vaccination, annotated with the anti-Spike Ab titers induced by d50 (**Figure 2C**). Patients who did not mount an appreciable Ab response to the vaccine (blue) partially segregated from patients with moderate-to-high Ab titers (brown-to-red) along PC2 (**Figure 2C**, left panel). The cytokines IL-8, MIP-1α, MCP-4, Eotaxin, TARC and VEGF mainly influenced PC2 (**Figure 2C**, right panel). Of note, patients C07 and C15, who were among those with the highest anti-Spike Ab titers, were two outliers along PC1 (**Figure 2C**, left panel, in red). PC1 was mainly influenced by the cytokines IFN-γ, IL-15, IP-10/CXCL10, SAA, IL-1Ra and IL-6 (**Figure 2C**, right panel).

Given their variability in regulating cytokine responses upon vaccination, patients in the high/moderate group were further divided into: (i) high responders (n=3, C07, C15 and C27), (ii) moderate responders (n=22) and compared to the low responder group (low; n=4; C16, C18, C25, C26) (**Figure 2A**). Median cytokine log2FC induced upon the 2^nd^ vaccination was compared among these 3 groups and depicted as radar plot (**Figure 2D**). High responders (red line) presented with the strongest activation of IFN-γ (>200x), IP-10/CXCL10 and IL-1Ra (~4x), IL-15, MIP1-β, IL-6 (~2x), IL-7, TSLP, CRP, TNF-a, IL-10 (~1.5x) and SAA (~8x). We also observed downregulation of Eotaxin, MCP-4 and the IL-17 family of cytokines. Moderate responders (green line) followed a similar pattern of cytokine regulation, but all the responses were diminished. Low responders (low; blue line) showed the weakest upregulation of the IFN-γ cytokine hub (~2x). A distinctive feature of their profile was the marked upregulation of MIP1-α and IL-8 (~8x) and the downregulation of IL-7.

To address the contribution if any of the wider Ab response range within the moderate group this group (n=22) was further divided into 2 subgroups using the geometric mean plus 0.5 log of the Ab titer as measure: Moderate high included 8 patients with anti-Spike antibody titers (geometric 5.1 log), while moderate low included 14 patients with anti-Spike antibody titers (geometric 4.1 log) (**Figure 2E**). Importantly, patients in the moderate_high group showed a more robust up-regulation of IFN-γ upon booster vaccination, in comparison to patients in the moderate_low group. However, such up-regulation was still significantly lower in comparison to high responder patients (50-fold reduced). A trend towards a higher up-regulation of CRP, SAA, IL-10 and IL-7 and higher downregulation of Eotaxin and MCP-4 was also observed in the moderate_high vs moderate_low group.

A similar analysis was also performed after the 1^st^ vaccination (**Supplementary Figure 2**). At d2, upregulation of IP-10/CXCL10, IFN-γ (~2x), IL-6 and IL-1Ra (~1.5x) was observed (**Supplementary Figures 2A, B**), which reached significance only in high/moderate responders, using a cut-off p value<0.05. No differences in the cytokine profile between d1 and d2 were observed in non-responders. Although differences in the vaccine-induced cytokine profile between the 3 groups of patients were also identified upon 1^st^ vaccination, the changes were overall smaller in comparison to 2^nd^ vaccination (**Supplementary Figure 2C****),** including the moderate subgroups (**Supplementary Figure 2D**).

Overall, these data indicated that the 2^nd^ vaccination in the patient cohort was associated with a cytokine signature, characterized by the acute upregulation of IFN-γ, IL-15 and IP-10/CXCL10. The signature also included IL-6 and IL-1Ra. The profile was enriched for cytokines regulating adaptive immunity (TNF-α, IL-7, IL-10 and TSLP), the chemokine MIP-1β and acute phase proteins in patients who developed high levels of anti-Spike Abs. Patients who failed to mount detectable humoral responses showed attenuated responses in the cytokine signature (IFN-γ, IL-15 and IP-10/CXCL10), concomitantly with a marked upregulation of MIP-1α, IL-8, MIP-1β, TNF-α and downregulation of IL-7.

### Correlation Between Cytokine Changes Induced by Vaccination in Patients

**Figure 3:**
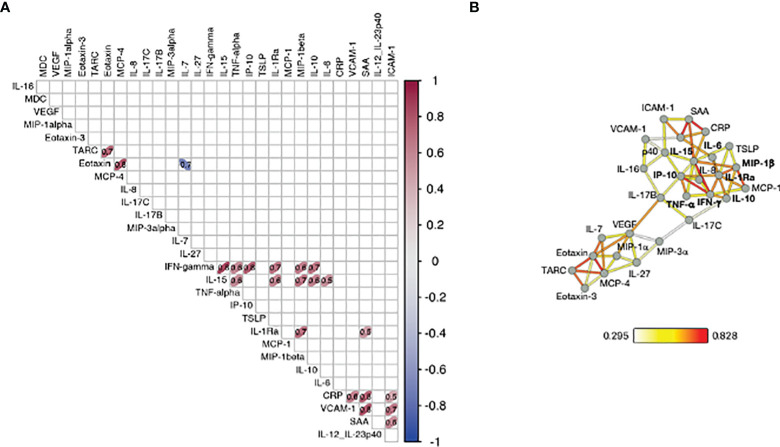
Correlation of cytokine changes upon 2^nd^ vaccination in patients. Pairwise correlations were calculated among the cytokines log2FC at d23 in comparison to d22, using the Spearman correlation coefficient (adjusted p value < 0.05). The analysis was performed for patients with moderate-to-high Ab responses to the vaccine (n = 25). **(A)** Correlation matrix for the 2^nd^ vaccination is plotted as heatmap. Spearman r values of correlations are indicated in the grid cells and ellipses identified significant correlations. The color and shape of ellipses correspond to the value of the Spearman correlation coefficient with red color indicating a positive correlation. **(B)** The network analysis represents the correlation profile among cytokine changes after 2^nd^ vaccination in patients. The color-coded edges represent the Spearman correlation coefficient (r) between two specific analytes, depicted as nodes.

Several vectors that influence PC1 in the loading plot (**Figure 2C**, right panel) group closely together suggesting a positive correlation among the variables they represent. To assess the inter-relationship of the vaccine-induced effects on different serum cytokines, we performed pairwise correlation analysis using the log2FC at d23 (2^nd^ vaccination) in the high/moderate responder subgroup (n=25). The correlation matrix was calculated and graphically depicted as heatmap, presenting associations above our cut-off for Spearman correlation coefficient corresponding to an adjusted p value <0.05 (**Figure 3A**). We identified strong correlated groups of cytokines and among the cytokine pairs that highly correlated were: (i) IL-15 and IFN-γ, TNF-α, IL-1Ra, MIP-1β, IL-10, IL-6; (ii) IFN-γ and IP-10/CXCL10, TNF-α, IL-1Ra, MIP-1β, IL-10; (iii) IL-1Ra and MIP-1β and SAA (**Figure 3A**). Additional clusters of correlations included the inflammatory mediators CRP, SAA, VCAM-1 and ICAM-1 and the chemokines TARC, Eotaxin and MCP-4.

Correlations between cytokine pairs induced upon vaccination were used to build the mediators network using Graphia (33) (**Figure 3B**). A central node featured the cytokines IFN-γ, IL-15 and IP-10/CXCL10 closely correlating with each other, a result consistent with a coordinated role of these cytokines in supporting both innate and adaptive immunity. This node also associated with a wider group of both inflammatory cytokines (including IL-6, IL-1Ra, MIP-1β, IL-10 and TNF-α) and mediators (SAA, CRP, VCAM-1, ICAM-1). We also identified a separate node including chemokines that showed a pattern of downregulation upon vaccination grouped together (Eotaxin, TARC and MCP-4). These correlations suggested a generic pattern of co-regulation as consequence of the vaccine-induced inflammation process.

Overall, the identified relationships strongly suggested a coordinated vaccine response driven by IL-15, IFN-γ, and IP-10/CXCL10 in patients, as we previously reported in a cohort of healthy vaccine recipients (21).

### Comparison of the Vaccine-Induced Cytokine Profile Between Patients and HCW

**Figure 4:**
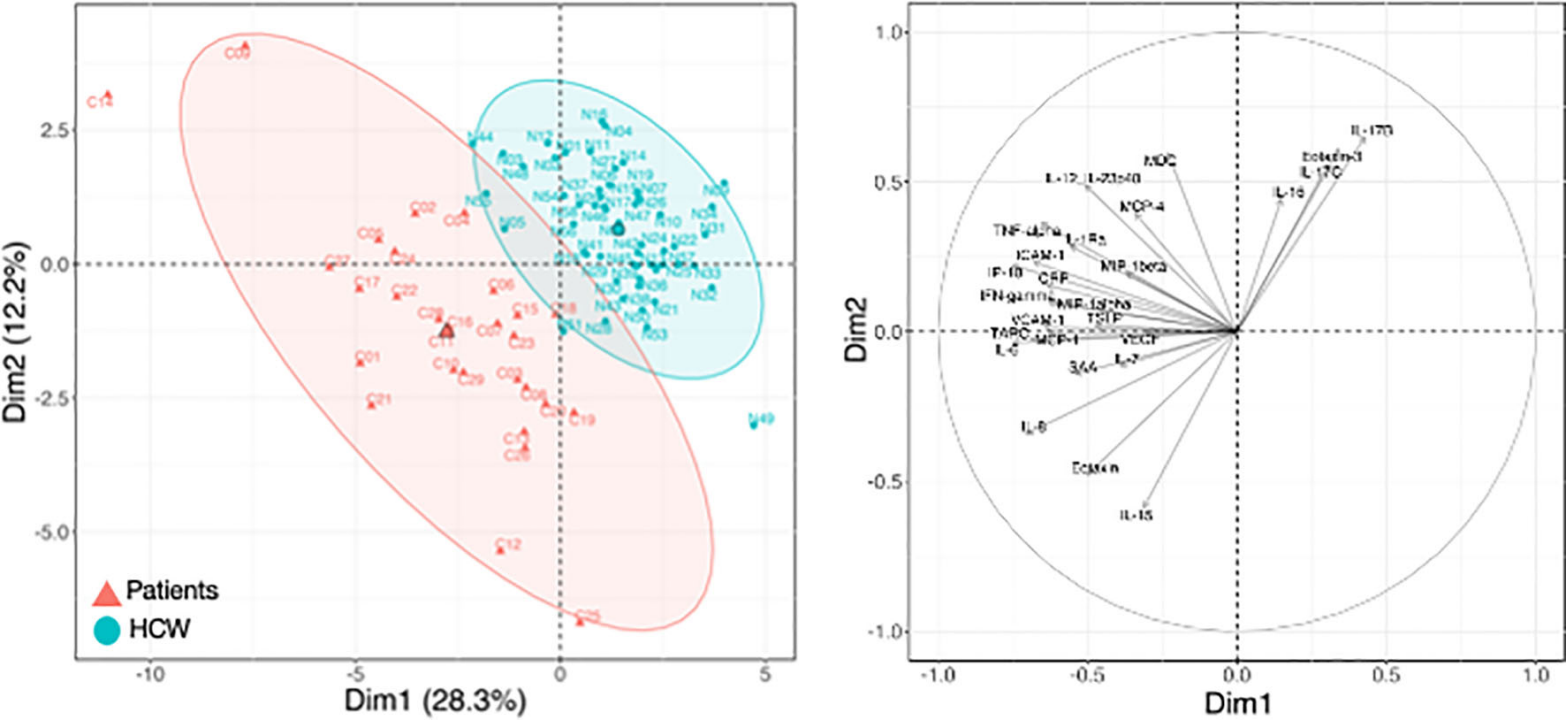
Cytokine levels at pre-vaccination in patients and HCW. Comparison of serum cytokine levels pre-vaccination (d1) between patients (n=29) and healthy volunteers [n = 57, as reported in (21)]. PCA plot (left panel) distinguishes patients (red triangle) from HCW (blue circle) based on their cytokine levels at day 1, capturing 28.3% and 12.2% of the dataset variance along PC1 and PC2, respectively. Median from each group is depicted as larger symbol. PCA loading plot (right panel) showed how strongly cytokines (depicted as grey vectors) influence both PC1 and PC2. The projection of the vectors on PC1 and PC2 corresponded to the loading value (weight) of the cytokine they represent.

**Figure 5:**
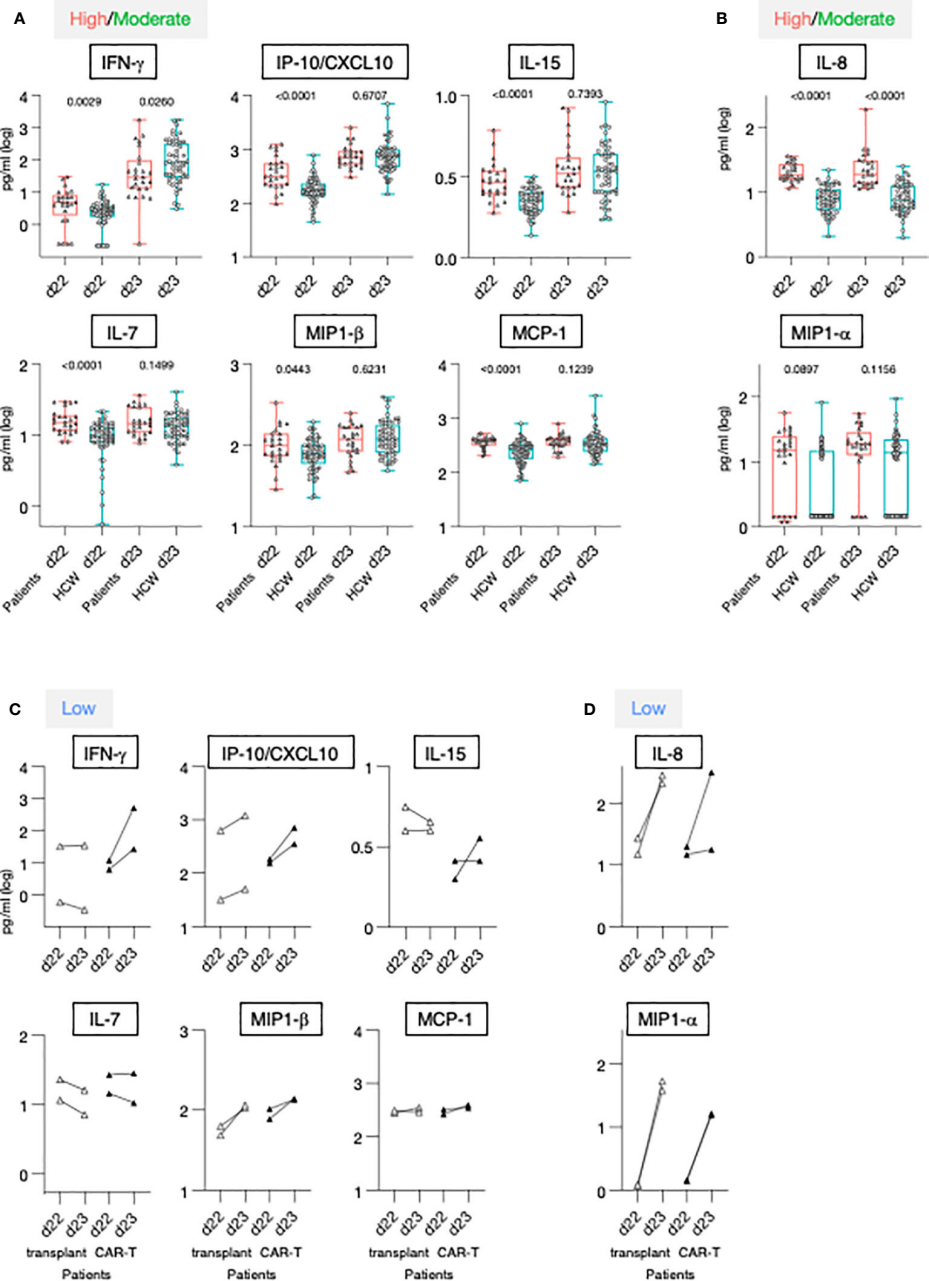
Serum cytokine levels after the 1^st^ and 2^nd^ vaccination in patients and HCW. **(A, B)** Cytokine and chemokine levels were measured over time using the MSD assay on the day of (d22) and at 24 hrs (d23) after 2^nd^ vaccination in 25 patients with moderate-to-high vaccine-induced Ab titer (red triangles) and 57 HCW [blue circles, as reported (21)]. Whisker plots show overtime log10 serum levels of the high/moderate group **(A)** IFN-γ, IP-10/CXCL10, IL-15, IL-7, MIP-1β, MCP-1 and **(B)** IL-8 and MIP-1α. Differences at each timepoint between these groups were analyzed using Mann-Whitney tests. **(C, D)** Cytokine levels detected in the 4 low responder patients, with Ab levels at or below threshold, are also plotted overtime, after the 2^nd^ vaccination (patients on medication lacking B cells, open triangles; patients received CAR-T cell therapy, filled triangles).

To further understand the response to vaccination, we compared the circulating levels of cytokines in the cohort of patients and HCW. Patients were characterized by significantly elevated levels of several cytokine pre-vaccination, both at d1 (**Figure 4**; **Supplementary Figure 3**) and at d22 (**Figure 5**; **Supplementary Figure 4**). PCA was performed on pre-vaccination cytokine levels (d1). The first two Principal Components (PC1 and PC2) captured ~40% of the variance in the entire dataset and allowed for the segregation of patients (red) and HCW (blue) into 2 distinct clusters (**Figure 4**, left panel). The PCA loading plot indicated that several inflammatory markers were the strongest determinants of the variance between the two cohorts (**Figure 4**, right panel). The cytokine levels at d1 for individual patients and the average for HCW vaccine recipients were represented in the heatmaps (**Supplementary Figure 3**). Overall, this analysis showed a different and much wider distribution among patients in comparison to HCW, regarding their baseline cytokine levels. Many cytokines associated with inflammation were upregulated in patients.

The 2^nd^ vaccination resulted in the upregulation of IFN-γ, IP-10/CXCL10 and IL-15 in both the patients in the high/moderate group and in HCW [see also **Figure 2** and (21)]. Importantly, at d23, IFN-γ levels were significantly lower in these patients in comparison to HCW. The patients were also characterized by elevated circulating levels of IP-10/CXCL10 and IL-15 at d22, but no significant differences for these cytokines were observed post-vaccination at d23 between the two cohorts (**Figure 5A**), indicating a diminished response to the vaccine in patients. Similarly, while significantly higher levels of IL-7, MIP-1β and MCP-1 were detected pre-vaccination (d22) in patients, serum concentrations comparable to those of HCW were detected after 2^nd^ vaccination (**Figure 5A**). Levels of the inflammatory cytokines IL-6, IL-1Ra, TNF-α, and IL-12/IL-23p40, and acute phase proteins CRP and SAA were significantly higher in patients both at d22 and d23 (**Supplementary Figure 4A**), despite their attenuated upregulation induced by vaccination. Analysis conducted on the four patients belonging to the non-responder group revealed heterogeneous and partial responses for some cytokines (**Figures 5C, D**; **Supplementary Figure 4B**). Vaccination induced the upregulation of IFN-γ in 2/4 patients; of IP-10/CXCL10 in 4/4; of IL-15 in 1/4; IL-7 in 0/4, MIP-1β in 4/4 and MCP-1 in 1/4, IL-6 in 4/4; IL-1Ra in 4/4, TNF-α in 4/4 and IL-12/23p40, CRP and SAA in 0/4. Different patterns were observed for IL-8 and MIP-1α. At d22, IL-8 serum levels were elevated in patients in comparison to HCW, while no significant difference was found for MIP-1α. The 2^nd^ vaccination induced the most significant upregulation in the four patients of the non-responder group (**Figures 5C, D**).

**Figure 6:**
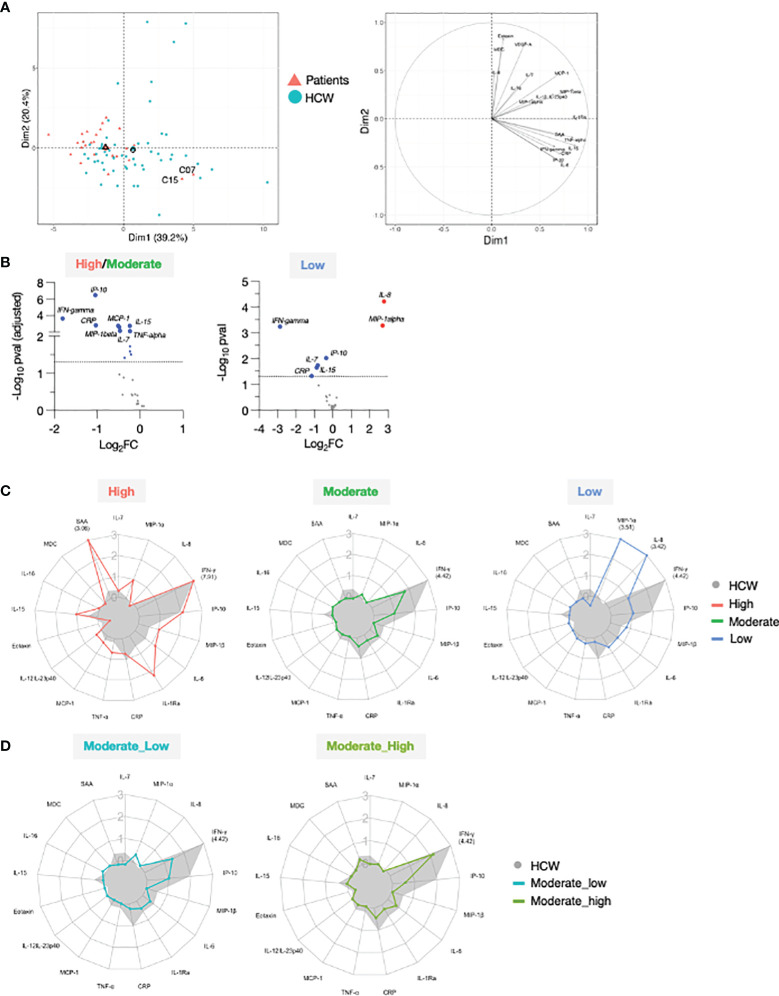
Comparison of serum cytokine and chemokine profile after 2^nd^ vaccination in patient and HCW vaccine recipients. **(A)** PCA plot (left) distinguish patients (red triangle) from healthy volunteers (blue circle) based on the cytokine log2FC at d23 in comparison to d22 (2^nd^ vaccination effect), capturing 39.2% and 20.4% of the dataset variance along PC1 and PC2, respectively. Median from each group is depicted as larger symbol. PCA loading plot (right) showed how strongly cytokines (depicted as grey vectors) influence both PC1 and PC2. The projection of the vectors on PC1 and PC2 corresponds to the loading value (weight) of the cytokine they represent. **(B)** Volcano plot depicts differentially regulated analytes after 2^nd^ vaccination (d23 compared to d22) between 25 patients in the high/moderate responder group and 57 HCW (left panel) and between 4 patients in the low responder group and 57 HCW (right panel). Blue dots indicate cytokines that were significantly less affected by 2^nd^ vaccination in patients in comparison to HCW. Significance was set at an adjusted p value < 0.05 (broken horizontal line) for the high/moderate group. A cut-off p value <0.05 was used for the group of low responders. **(C)** Radar plots compared the median log2FC for each cytokine after 2^nd^ vaccination (d23 compared to d22) between HCW (n = 57, filled grey area) and patients with high (left panel; red, n = 3), moderate (middle panel; green, n = 22), and low (right panel; blue, n = 4) anti-Spike Ab titers measured at d50. Plot axis is truncated at log2FC=3, with values exceeding this cutoff shown in parentheses (high responders: IFN-γ=7.91; SAA=3.06; HCW: IFN-γ=4.42). **(D)** Patients in the moderate group were further separated into moderate_high (n = 8) and moderate_low (n = 14). Radar plot compared the median log2FC for each cytokine after 2^nd^ vaccination (d23_22) between HCW and patients in the moderate_high and moderate_low groups.

To compare the overall vaccine-induced cytokine signature upon 2^nd^ vaccination between patients and HCW, we performed PCA on the cytokine log2FC at d23 over d22 (**Figure 6A**, left panel). PC1 and PC2 accounted for ~60% variance of the entire data set. Most of the patients fell in the PCA upper left quadrant, while HCW were more widely distributed, with many segregating from patients along PC1 (upper and lower right quadrants). A PCA loading plot demonstrated that some specific mediators strongly contributed to the differences observed between the two cohorts of vaccine recipients. Segregation along PC1 was mainly driven by changes in a group of closely associated molecules that included IFN-γ, IL-15, IP-10/CXCL10, IL-6, TNF-α, CRP and SAA (**Figure 6A**, right panel). Interestingly, C15 and C07 who were among the patients who mounted high humoral responses upon vaccination (**Figures 1**, **2C**) were outliers along PC1 and clustered closely to HCW.

We performed differential expression analysis on the log2FC at day 23 between patients and HCW. This comparison was performed by separating patients into high/moderate (n=25) and non-responder (n=4) groups, as in **Figure 2**. Important quantitative difference in the ability to respond to the mRNA vaccine between these cohorts were identified. In agreement with **Figure 5**, patients in the high/moderate responder group were characterized by a diminished upregulation of IFN-γ (~4x), IP-10/CXCL10, CRP (~2x), IL-15, IL-7, TNF-α, MCP-1 and MIP-1β (~1.5x) (**Figure 6B**, left panel), suggesting an overall attenuated systemic cytokine response to vaccination. A diminished vaccine-induced effect on IFN-γ, IP-10/CXCL10, IL-15, IL-7, and CRP was also observed in patients in the non-responder group in comparison to HCW. Of note, the effect on IFN-γ was diminished by ~8x in the patient group. Importantly, in comparison to HCW, these patients showed a more pronounced upregulation of MIP-1α and IL-8 (~4x) (**Figure 6B**, right panel). A comparison with HCW was also performed further separating the patients into 3 groups based on their anti-Spike Ab levels at d50, as described in **Figure 2** (**Figure 6C**). High responders (red) showed a partially enhanced systemic cytokine response than the one elicited by HCW, with a greater upregulation of IFN-γ, IL-15, and several other chemokines and inflammatory mediators, including IL-6, IL-1Ra, TNF-α, and SAA (**Figure 6C**, left panel). A similar regulation of IP-10/CXCL10 and IL-7 was found between these two groups. Moderate responders (green) followed a similar pattern of cytokine regulation as HCW (grey area), but all responses were diminished (**Figure 6C**, middle panel). In low responders (blue), the systemic response to vaccination showed a marked attenuation in the IFN-γ/IP-10/CXCL10/IL-15 signature and was dominated by IL-8 and MIP-1α (**Figure 6C**, right panel). A similar analysis was also performed subdividing the moderate group into moderate_high and moderate_low, as described for **Figure 2** was performed (**Figure 6D**). In both groups, all cytokine responses to vaccination were diminished in comparison to HCW. The most striking difference between moderate_high and moderate_low group was in the up-regulation of IFN-γ. Patients in the moderate_high group were characterized by 2-fold increase in the IFN-γ upregulation in comparison to patients in the moderate_low group, although this effect was still 5-fold reduced in comparison to HCW.

Overall, these data showed that, in this cohort of patients in comparison to HCW, BNT162b2 mRNA vaccination resulted in a diminished IFN-γ/IP-10/CXCL10/IL-15 response. Importantly, patients with different anti-Spike Ab titers at d50 could be distinguished based on their vaccine-induced modulation of cytokines involved in innate and adaptive immunity, underlying the opportunity to identify cytokine changes as early biomarker of successful vaccination and to improve our understanding of the mechanisms leading to vaccine efficacy and increased protective immunity, especially in immunocompromised individuals.

### Biomarkers of Effective Vaccination

The variability of the responses to vaccination observed in patients allowed for the examination of the relationships between alterations in serum cytokines and the levels of anti-Spike Ab levels detected either at d22 (3 weeks after vaccination 1) or at d50 (4 weeks after vaccination 2), with the aim to identify biomarkers of successful vaccination response. We have previously found that both IFN-γ and IL-15 log2FC induced by 2^nd^ vaccination in HCW correlated with the anti-Spike Ab levels detected at day 36 in healthy volunteers (21).

**Figure 7:**
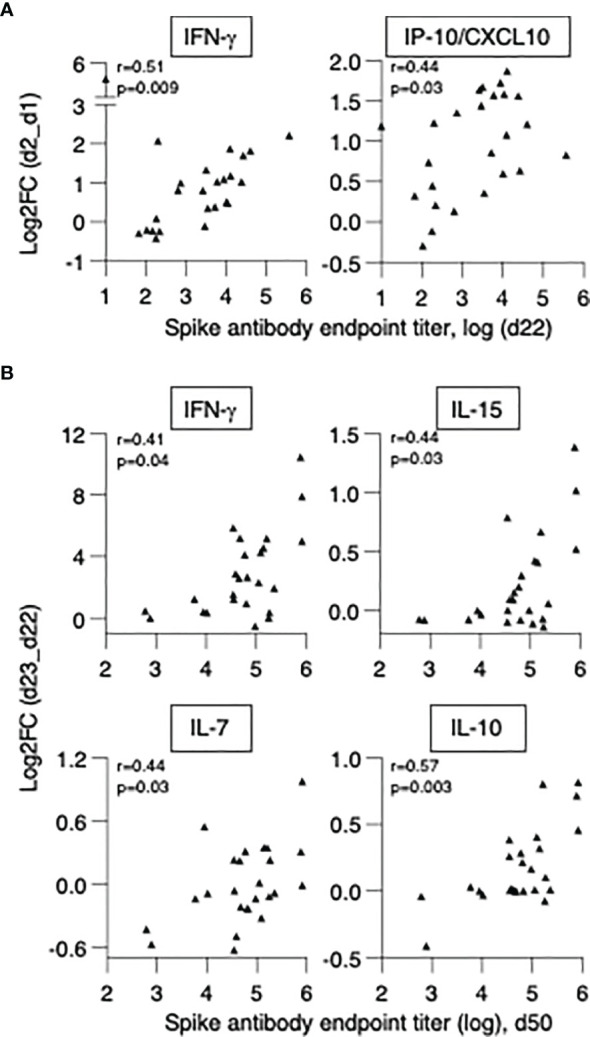
Biomarkers of effective vaccination. Correlations of log2FC after the 1^st^ vaccination (d2_d1) of **(A)** IFN-γ and IP-10/CXCL10 and levels of anti-Spike Ab measured at d22. Correlations of log2FC after the 2^nd^ vaccination (d23_d22) of **(B)** IFN-γ, IL-15, IL-7, IL-10 and levels of anti-Spike Ab measured at d50. The analysis was performed on patients in the high/moderate group (n = 25). Spearman r and p values are given.

We first investigated whether cytokine changes induced by the 1^st^ vaccination could predict Ab development in patients. A positive correlation was found between anti-Spike Ab measured (d22) and log2FC (d2) of IFN-γ (r=0.51, p=0.009) and IP-10/CXCL10 (r=0.44, p=0.028) (**Figure 7A**). We also investigated correlations between cytokine changes induced by the 2^nd^ vaccination and anti-Spike Ab titers, using the cohort of 25 patients with moderate-to-high Ab responses. We found positive Spearman correlations of anti-Spike Ab titers at d50 with log2FC of: IFN-γ (r=0.41, p=0.04), IL-15 (r=0.44, p=0.027), IL-7 (r=0.44, p=0.029) and IL-10 (r=0.57, p=0.003) (**Figure 7B**). Additional correlations are shown in **Supplementary Table 3**. Together, these results suggested a coordinate response to the BNT162b2 mRNA vaccine and highlighted the important role of rapid innate responses to vaccination in shaping adaptive immunity.

In conclusion, in patients with hematological malignancies who underwent cell therapies, the ability to acutely upregulate IFN-γ and IP-10/CXCL10 at the priming vaccination and IFN-γ, IL-15, IL-7 and IL-10 upon booster vaccination were predictive of successful Ab development. These data support the use of this signature as biomarker for effective SARS-CoV-2 Ab responses upon BNT162b2 mRNA vaccination.

## Discussion

This report provides a comparative analysis of the innate and immunological responses induced by a 2-dose BNT162b2 mRNA vaccination in patients with hematological malignancies, who received cell therapies, and a cohort of healthy vaccine recipients. This study reveals differences in anti-SARS-CoV-2 Spike Ab titers and the cytokine signature elicited upon BNT162b2 mRNA vaccination in the two cohorts. The patients showed heterogeneous adaptive and innate responses with overall lower humoral and reduced innate cytokine responses to vaccination. Our analysis identified a cytokine signature (IFN-γ, IP-10/CXCL10, IL-15) as biomarker for effective SARS-CoV-2 Ab responses upon BNT162b2 mRNA vaccination. Systems immunology approaches represent a tool to identify innate signatures associated with protective adaptive responses to vaccines. The pattern of responses we described in this report offer novel prognostic approaches for potentiating the effectiveness of COVID-19 vaccination in transplant patients with hematological malignancies. Identification of early immunization biomarkers is likely to improve our understanding of the mechanisms leading to vaccine efficacy and contribute to efforts towards increased protective immunity, especially in immunocompromised individuals.

The Spike Ab response in the patient cohort showed a proportional lower ability of cross-recognition of different VOC Spike-RBD proteins (Beta, Delta, Omicron) and lower ability to mediate neutralization compared to the wildtype WA1 strain. Importantly, although a significant correlation between binding Ab levels and their neutralization ability was found for both the wildtype WA1 and Delta variant, both the patient and HCW cohorts failed to neutralize the Omicron Spike pseudotyped virus, except for a few vaccinees who had very high anti-Spike Ab titers. The recognition of Beta- and Omicron-RBD was significantly lower due to critical changes within RBD. These data are in-line with on our previous report of poor neutralization of Beta-Spike pseudotyped viruses by the HCW cohort (27), and here, we further report a lack of neutralization of Omicron. These data are in agreement with recent reports showing drastic reduction of Omicron neutralization after two BNT162b2 mRNA vaccinations in HCW (34–36). Thus, additional vaccination doses are necessary to augment Spike immune responses supported by longer time since transplantation and this will also provide the necessary boost to increase NAb against Omicron and other possible VOC (35–39).

We previously showed that BNT162b2 mRNA vaccination in healthy volunteers elicited a cytokine signature featuring IFN-γ, IP-10/CXCL10 and IL-15 (21). Transient increases in IL-15 and IFN-γ levels early after booster vaccination correlated with anti-Spike Ab levels, supporting their possible use as biomarkers of successful vaccination. In this study, hematopoietic stem cell transplant and/or CAR-T cell recipients showed heterogeneous responses to the vaccination and were classified into three categories (high, moderate, and low/non-responders), based on their anti-Spike Ab levels detected at d50. Patients who mounted humoral responses upon vaccination (high/moderate groups) were characterized by significantly elevated systemic levels of IFN-γ, IL-15, and IP-10/CXCL10, at 24 hrs after the 2^nd^ vaccine dose. Three patients who developed high levels of anti-Spike Ab presented with the highest upregulation of these cytokines and were characterized by a vaccine-induced innate signature similar, to that of HCW. Their signature was enriched for cytokines involved in the development and modulation of adaptive responses (IL-7, TNF-α, IL-10), mediators involved in inflammatory processes, including IL-1Ra, IL-6, IL-12/IL-23p40, and acute phase proteins SAA and CRP. In patients with moderate Ab responses, the vaccine-induced cytokine signature was overall attenuated in comparison to HCW, with a marked 4x reduction in IFN-γ upregulation. Patients who failed to mount humoral responses showed the weakest IFN-γ increase, upregulation of MIP-α and IL-8 and downregulation of IL-7.

A role for IFN-γ and IFN-dependent antiviral immunity upon BNT162b2 mRNA vaccination has been previously identified (40, 41). Booster vaccination resulted in the emergence of a myeloid population with an enriched interferon-stimulated gene program and induced higher concentration of circulating IFN-γ. The authors hypothesized that natural killer cells, tissue-resident T cells or innate lymphoid cells at the site of vaccination or draining lymph nodes, rather than PBMC, could be the potential source of the induced IFN-γ. Our findings identified changes in IFN-γ as an important effect of BNT162b2 mRNA vaccination, both in hematopoietic stem cell transplant or CAR-T cell recipients and in HCW. In the moderate/high groups, we found a significant correlation between anti-Spike Ab titers and changes in IFN-γ and IP-10/CXCL10 levels after the 1^st^ vaccine dose, and between anti-Spike Ab titers and changes in IFN-γ and IL-15 levels after the 2^nd^ vaccine dose, suggesting that the cytokine signature consisting of IFN-γ, IL-15, and IP-10/CXCL10 could serve as a biomarker of effective vaccination in vaccinated transplant patients with hematological malignancies, as previously reported for HCW (21).

It is plausible that the immunocompromised status of the patients may impact their ability to both mount anti-Spike Ab, as reported by other studies, and modulate their systemic cytokine profile upon vaccination. At pre-vaccination (d1), the transplanted patient cohort had higher serum levels of several cytokines compared to HCW, indicative of a possible hyperactive or dysfunctional immune system. Assessment of cytokine levels at baseline alone was not sufficient to predict the positive development of humoral responses to vaccination.

Although our patient cohort showed lower anti-Spike Ab titers than healthy individuals, a significant positive correlation was found between anti-Spike Ab titers and time since transplantation, indicating that vaccine-relevant reconstitution improved over time, and it may take ~2 years. Several studies have investigated the efficacy of COVID-19 vaccination in patients with different type of hematological malignancies, reporting overall low and mixed seroconversion rates. The ability to mount humoral responses greatly varied depending on the type of cancers, patients’ age, and treatment. Treatments including anti-CD20, anti-CD38, and anti-BCMA antibodies, CAR-T cells, chemotherapy, corticosteroids, and stem cell transplantation (SCT) negatively affect lymphocyte numbers and function, and result in poor responses to the vaccine. In agreement with these findings, the patient cohort enrolled in our study developed significantly lower and more heterogeneous anti-Spike Ab titers after each vaccine dose compared to HCW. Both cohorts had significant increases in Ab titers after the vaccine booster administration, characteristic of an anamnestic response. Fourteen percent of patients (4/29) failed to develop antibodies even after the 2^nd^ dose. Of these, two patients had recently received CAR-T cell therapy 0.3-1.2 years before vaccination, and two patients were on medications known to impact B cell function. In support of our results, Herishanu, et al. (42) and Terpos et al. (43) found that CLL patients who had received anti-CD20 therapy up to 12 months prior to BNT162b2 mRNA vaccination mount a low humoral response. Tamari et al. (3) reported that immune recovery following allogeneic hematopoietic cell transplant in patients with hematological malignancies correlated with positive responses to COVID-19 vaccination. The time interval between cellular therapy and vaccine administration was a strong predictor of successful vaccination, with the highest humoral responses observed in patients vaccinated one year or later after transplantation. Additional studies in patients with hematological cancers found an association between higher CD19 levels and humoral response to the vaccine (14, 15). Together with our data on the significant positive correlation between anti-Spike Ab titers and time since transplant, these findings indicated the need for several years for vaccine-relevant immune reconstitution. This does not negate the current recommendation of the American Society of Transplantation and Cellular Therapy (ASCT) that SARS-CoV-2 vaccination should be offered as early as three months following SCT or CAR-T cell therapy.

Despite the lower antibody titers, patients with hematological malignancies still benefit from vaccination due to their preserved ability to mount specific cellular immune responses. T cells have been associated with viral clearance and disease pathogenesis attenuation. Several studies reported the development of strong anti-spike T cell responses upon vaccination in patients with severe humoral immune deficiencies, including patients treated with anti-CD20 antibody and CAR-T cell therapies targeting CD19 and CD22 (44, 45). These studies also highlighted the possibility to refine strategies to further increase T cell mediated immunity in individuals with B cell count and function anomalies.

Although our study is limited by its cohort size (n=29) and heterogeneity of disease and cell therapy treatment, our data support the vaccine-induced cytokine signature previously reported for healthy vaccinees and identify an immunological pathway involving IL-15, IFN-γ and IP-10/CXCL10 for optimal response to vaccination, also in immunocompromised individuals. Although we recognize the relatively small size of the patient cohort which nevertheless allowed to draw statistically validated conclusions.

Overall, our data revealed a heterogeneous spectrum of responses to BNT162b2 mRNA vaccination in patients with hematological malignancies who received cell therapies ranging from individuals unable to produce IFN-γ and IL-15 and unable to mount an Ab response to those whose innate and adaptive responses were similar to healthy individual. The pattern of responses described in this report offer novel prognostic approaches for potentiating the effectiveness of COVID-19 vaccination in transplant patients with hematological malignancies.

## Data Availability Statement

The original contributions presented in the study are included in the article/**Supplementary Files**. Further inquiries can be directed to the corresponding author.

## Ethics Statement

The studies involving human participants were reviewed and approved by Ethics Committee of Alexandra General Hospital (Ref No. 15/23 December 2020) and Evangelismos General Hospital (Ref No is 26/01-20-2021). The patients/participants provided their written informed consent to participate in this study.

## Author Contributions

ET, M-AD, GP, BF conceived and designed the study; MP, IT, MV, ID, IB, SG collected and processed patient data and samples; CB, MR, MA, JB, SD, BF, GP performed experiments and data analysis; CB, MA, MR, SD, BF, GP verified the underlying data; CB, MA, MR, AU, BF visualization; CB, BF, GP, ET drafted the manuscript. All authors reviewed and edited the manuscript and gave final approval for the submitted version.

## Funding

This work was supported by funds from the Intramural Research Program, National Institutes of Health, National Cancer Institute, Center for Cancer Research to GP. and BF. The funders had no role in the experimental design, collection of data or writing the paper.

## Author Disclaimer

The content of this publication does not necessarily reflect the views or policies of the Department of Health and Human Services, nor does mention of trade names, commercial products, or organizations imply endorsement by the U.S. Government.

## Conflict of Interest

Author MA was employed by Leidos Biomedical Research, Inc.

The remaining authors declare that the research was conducted in the absence of any commercial or financial relationships that could be construed as a potential conflict of interest.

## Publisher’s Note

All claims expressed in this article are solely those of the authors and do not necessarily represent those of their affiliated organizations, or those of the publisher, the editors and the reviewers. Any product that may be evaluated in this article, or claim that may be made by its manufacturer, is not guaranteed or endorsed by the publisher.
