# Supplementary material for: Reduced Antibodies and Innate Cytokine Changes in SARS-CoV-2 BNT162b2 mRNA Vaccinated Transplant Patients With Hematological Malignancies
Source: Front Immunol. 2022 May 25;13:899972. doi: 10.3389/fimmu.2022.899972 (PMC9174567; doi:10.3389/fimmu.2022.899972)
Supplement: Supplementary file 1 [file DataSheet_1.docx]

**Supplemental Material**

**Supplementary Table 1: Patient cohort overview**

| Patient ID | Hematological Malignancy | Cellular therapies | Donor Type^1^ | Condi-tioning^2^ | Active Graft vs Host Disease | Rituximab treatment | Current immune-suppression | Current therapy for hematological malignancy | B cell (CD19+/υl) within 6 months prior to vaccination | Anti-Spike Ab endpoint titer (log), d50 | COVID-19 (+) post vaccin-ation (time, months) |
| --- | --- | --- | --- | --- | --- | --- | --- | --- | --- | --- | --- |
| C01 | myelodysplastic syndrome | allogeneic | Double UCB | MA | no | no | no | no | 717 | 3.8 | no |
| C02 | Acute Myeloid Leukemia | allogeneic | MUD | MA | yes | no | Mycophenolate Mofetil | no | 213 | 5.3 | no |
| C03 | Acute Myeloid Leukemia | allogeneic | MSD | MA | no | no | no | no | nd | 4.8 | yes (9 mo) |
| C04 | Acute lymphocytic leukemia | allogeneic | Double UCB | MA | no | no | no | no | 1423 | 4.8 | no |
| C05 | Chronic Myeloid Leukemia | allogeneic | MUD | RIC | no | yes | no | no | 615 | 5.4 | yes (9 mo) |
| C06 | Acute myeloid leukemia | allogeneic | Double UCB | MA | no | yes | no | no | 199 | 4.8 | No |
| C07 | Acute myeloid leukemia | allogeneic | MUD | MA | no | no | no | no | 1055 | 5.9 | No |
| C08 | Mycosis Fungoides | allogeneic | MMUD | RIC | yes | no | cyclosporine | no | 1325 | 5 | No |
| C09 | Hodgkin lymphoma | Autologous (2013) + allogeneic | MMUD | RIC | no | yes | no | no | 289 | 4.5 | no |
| C10 | acute lymphocytic leukemia | allogeneic | MMUD | MA | yes | no | Mycophenolate Mofetil | no | 283 | 4.5 | No |
| C11 | acute myeloid leukemia | allogeneic | MMUD | MA | yes | no | Mycophenolate Mofetil | no | 280 | 5.1 | No |
| C12 | chronic lymphocytic leukemia | allogeneic | MUD | RIC | yes | no | low dose steroids | no | 97 | 2.9 | No |
| C13 | acute myeloid leukemia | allogeneic | MMUD | MA | yes | no | cyclosporine | no | nd | 4.7 | No |
| C14 | myelodysplastic syndrome /acute myeloid leukemia | allogeneic | MSD | RIC | no | no | cyclosporine | no | 4 | 2.8 | No |
| C15 | Acute Myeloid Leukemia | allogeneic | MUD | MA | no | yes | no | no | nd | 5.9 | No |
| C16 | Diffuse large B-cell lymphoma | Autologous (2019) + CAR-T cells |  | N/A | n/a | no | n/a | no | 0 | 1 | No |
| C17 | acute myeloid leukemia | allogeneic | MUD | MA | yes | yes | Mycophenolate Mofetil | no | nd | 5.3 | No |
| C18 | primary mediastinal B-cell lymphoma | CAR-T cells |  | N/A | n/a | yes | n/a | no | 0 | 1 | yes (4 mo) |
| C19 | Non-Hodgkin lymphoma | allogeneic | Haplo | MA | no | no | no | no | 520 | 4.7 | No |
| C20 | myelodysplastic syndrome | allogeneic | MSD | MA | yes | no | tacrolimus, low dose steroids | no | 56 | 4.5 | No |
| C21 | multiple myeloma | autologous | Auto | N/A | n/a | no | lenalidomide for MM | yes | nd | 4 | no |
| C22 | acute myeloid leukemia | allogeneic | MUD | MA | no | no | no | no | 860 | 4 | No |
| C23 | Non-Hodgkin lymphoma | autologous | Auto | N/A | n/a | yes | no | no | nd | 5.1 | No |
| C24 | Hodgkin lymphoma | allogeneic | MSD | RIC | no | no | no | no | nd | 5.2 | No |
| C25 | Myelofibrosis | allogeneic | MUD | RIC | no | no | tacrolimus, steroids | no | 430 | 1.7 | No |
| C26 | acute lymphocytic leukemia | allogeneic | MMUD | MA | yes | yes | Mycophenolate Mofetil, Ruxolitinib | no | 3 | 1 | yes (8 mo) |
| C27 | acute myeloid leukemia | allogeneic | MUD | MA | no | no | no | no | 303 | 5.9 | No |
| C28 | acute myeloid leukemia | allogeneic | MUD | MA | no | no | no | no | nd | 5.1 | No |
| C29 | acute myeloid leukemia | Autologous (2005) +allogeneic | Double UCB | MA | no | no | no | no | nd | 4.6 | No |

^1^ UCB: Umbilical Cord Blood; MSD: Matched Sibling Donor; Auto: Autologous; MUD: Matched Unrelated Donor; MMUD: Mismatched Unrelated Donor

^2^MA: Myeloablative; RIC: Reduced Intensity Conditioning

**Supplementary Table 2. Cytokines and chemokines (MSD platform)**

| **Measurable Analytes (N=29)** | **Analytes below detection threshold (N=18)** |
| --- | --- |
| CRP (C-Reactive Protein) | GM-CSF |
| Eotaxin (CCL11) | IL-1a |
| Eotaxin-3 | IL-1b |
| ICAM-1 | IL-2 |
| IFN-γ | IL-3 |
| IP-10 (CXCL10) | IL-4 |
| IL-1Ra | IL-5 |
| IL-6 | IL-9 |
| IL-7 | IL-12p70 |
| IL-8 | IL-13 |
| IL-10 | IL-17A |
| IL-12/IL-23p40 | IL-17A/F |
| IL-15 | IL-17D |
| IL-16 | IL-21 |
| IL-17B | IL-22 |
| IL-17C | IL-23 |
| IL-27 | IL-31 |
| MCP-1 (CCL2) | TNF-b |
| MCP-4 |  |
| MDC (CCL22) |  |
| MIP-1α (CCL3) |  |
| MIP-1β (CCL4) |  |
| MIP-3α (CCL20) |  |
| SAA (serum amyloid A) |  |
| TARC (CCL17) |  |
| TNF-α |  |
| TSLP |  |
| VCAM-1 |  |
| VEGF |  |

**Supplementary Table 3. Correlations of anti-Spike Ab at d50 and**

**cytokines log2 fold changes upon 2^nd^ vaccination (d23_d22)**

|  | Spearman r correlation  coefficient | *p* value* |
| --- | --- | --- |
| CRP | 0.35 | 0.085 |
| **Eotaxin** | **-0.43** | **0.033** |
| Eotaxin-3 | -0.17 | 0.405 |
| ICAM-1 | 0.35 | 0.085 |
| **IFN-γ** | **0.41** | **0.04** |
| IL-1Ra | 0.35 | 0.09 |
| IL-6 | 0.38 | 0.06 |
| **IL-7** | **0.44** | **0.029** |
| IL-8 | -0.37 | 0.072 |
| **IL-10** | **0.57** | **0.003** |
| **IL-12/IL-23p40** | **0.4** | **0.046** |
| **IL-15** | **0.44** | **0.027** |
| IL-16 | -0.05 | 0.824 |
| IL-17B | -0.26 | 0.204 |
| **IL-17C** | **-0.41** | **0.043** |
| IL-27 | 0.27 | 0.187 |
| IP-10/CXCL10 | 0.3 | 0.149 |
| MCP-1 | 0.35 | 0.09 |
| MCP-4 | -0.18 | 0.39 |
| MDC | 0.04 | 0.844 |
| MIP-1α | -0.02 | 0.943 |
| MIP-1β | 0.33 | 0.104 |
| MIP-3α | 0.142 | 0.580 |
| **SAA** | **0.55** | **0.043** |
| TARC | -0.22 | 0.298 |
| **TNF-α** | **0.45** | **0.024** |
| TSLP | 0.32 | 0.121 |
| VEGF | -0.05 | 0.797 |
| VCAM-1 | 0.37 | 0.066 |

* Correlations were determined using a cut-off for a Spearman correlation coefficient

corresponding to a p value <0.05 (non-adjusted for multiple comparison)

**Supplementary Figure 1.** Analysis of vaccine-induced anti-Spike Ab. **(A)** Comparison of Spike Ab titers at day 50 of patients (n=29) and HCW (n=55) and HCW subgroups (n=30 and n=20), as they were used in Fig. 1C to F). (**B**) Correlation of anti-WA1 Spike Ab endpoint titers (log) measured at day of 2^nd^ vaccination (d22) and one month later (d50) in patients (left panel) and HCW (right panel) vaccine recipients. The three patients with very high Ab titers are deonted with square symbols throughout. **(C)** Side-by-side comparison of Spike-RBD Ab titers of patient and HCW cohorts at day 50. Data are from Fig. 1C. (**D**) Correlation of Ab endpoint titers to Delta Spike-RBD and Delta NAb (ID50) titers of patients (left panel, n=20) and HCW (right panel, n=20) vaccinees. (**E**) Correlation of NAb ID50 titers of Delta and WA1(D614G) in patients (left panel, n=20) and HCW (right panel, n=20) vaccinees. Sample selection is described in Fig 1D. Spearman r and p values are given for all correlations.

**Supplementary Figure 2.** Cytokine and chemokine profile in patients after 1^st^ vaccination. Serum cytokine levels were measured before (d1) and 24 hours (d2) after the 1^st^ vaccination using the MSD platform. (**A**) Heatmap depicted log2FC for 29 analytes at d2 in comparison to baseline levels at d1. Heatmap is annotated with anti-Spike Ab titer measured at d50 and years since cell therapy (transplantation and CAR-T) for each patient. Patients were separated into two groups based on their ability to mount Ab responses to the vaccination: high/moderate responders (left heatmap) and low responders (right heatmap). Patients were clustered based on their overall cytokine/chemokine profile. (**B**) Volcano plot of data shown in Panel A depicts differentially expressed cytokines upon 2^nd^ vaccination at d2 in comparison to d1 for patients in the high/moderate group (n=25). Red dot indicates significant upregulation (p value <0.05 represented by the broken horizontal line). (**C**) Patients were separated into 3 groups based on anti-Spike Ab titers measured at d50 (low in blue, n=4; moderate in green, n=22; high in red, n=3). Radar plot compared the median log2FC for each cytokine after the 1^st^ vaccination (day 2_1) among the three groups. (**D**) Patients in the moderate group were further separated into moderate_high (n=8) and moderate_low (n=14). Radar plot compared the median log2FC for each cytokine after 2^nd^ vaccination (d2_1) between the subgroups.


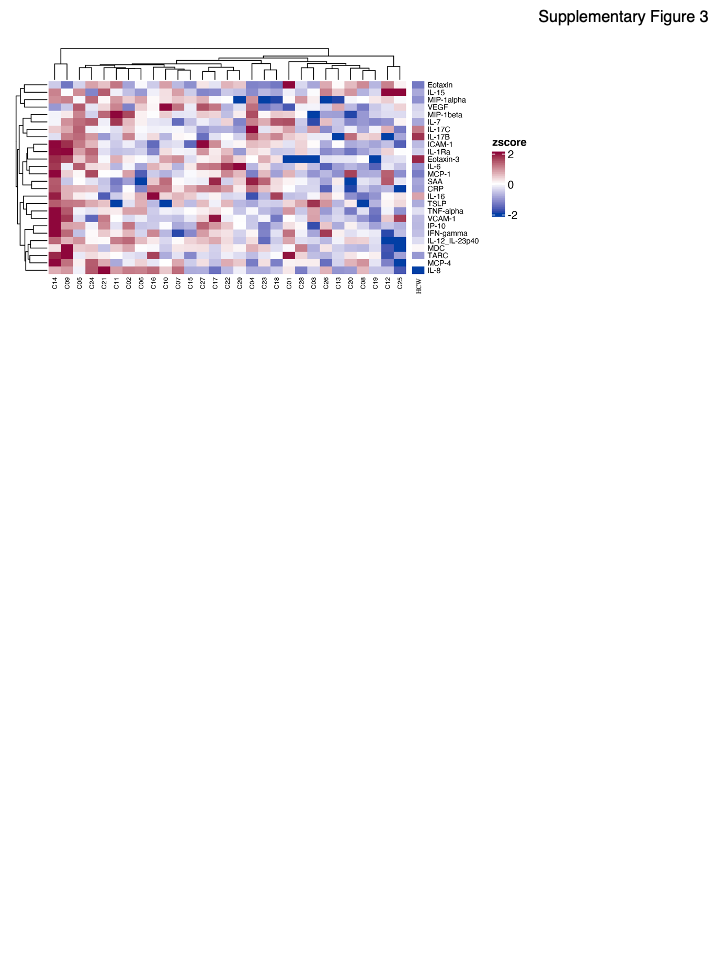

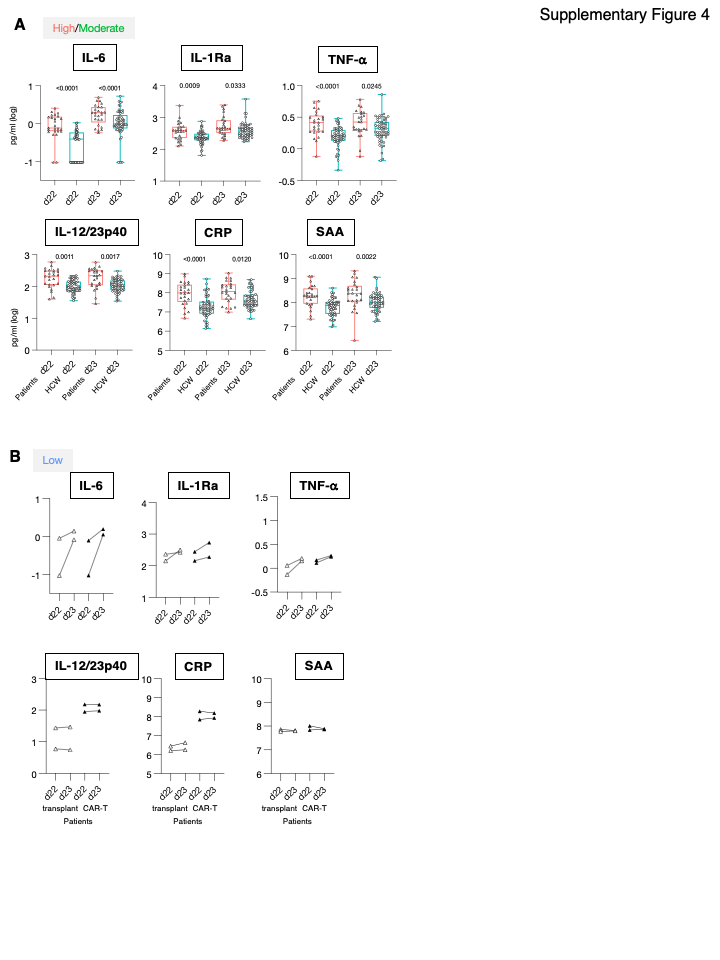


**Supplementary Figure 3.** Comparison of cytokine levels at pre-vaccination between patients and HCW. Serum levels were measured before (d1) vaccination using the MSD platform in both patients (n=29) and HCW (n=57). Heatmaps, represented as Z-score centered and rescaled, depict the cytokine levels for individual patients (left) and the average of HCW (right).

**Supplementary Figure 4**. Additional serum cytokine/chemokine levels affected by vaccination in patients and HCW. Cytokine levels were measured over time using the MSD assay in 25 patients with (**A**) moderate-to-high vaccine-induced Ab titer (red triangles) and 57 HCW (blue circles). Whisker plots show overtime log10 serum levels of seven selected analytes involved in inflammation (cytokines and acute phase proteins). Differences at each timepoint between these groups were analyzed (Mann-Whitney test). **(B**) Cytokine levels detected after 2^nd^ vaccination in the four low responder patients are also plotted overtime. Two patients are on medication (affecting B cell development and function; open triangles) and two patients received CAR-T cell therapy (filled triangles).
